# Supplementary material for: A magnetic multi-layer soft robot for on-demand targeted adhesion
Source: Nat Commun. 2024 Jan 20;15:644. doi: 10.1038/s41467-024-44995-9 (PMC10799857; doi:10.1038/s41467-024-44995-9)
Supplement: Supplementary file 1 — Supplementary Information [file 41467_2024_44995_MOESM1_ESM.pdf]

Supplementary Information

A magnetic multi-layer soft robot for on-demand targeted adhesion

Ziheng Chen[1,2,3+], Yibin Wang[2,3+], Hui Chen[2,3], Junhui Law[2,4], Huayan Pu[1], Shaorong Xie[5], Feng Duan[6], Yu Sun[4], Na Liu[1*] and Jiangfan Yu[2,3,7*]

[1]School of Mechatronics Engineering and Automation, Shanghai University, Shanghai 200444, China.

[2]School of Science and Engineering, The Chinese University of Hong Kong, Shenzhen 518172, China.

[3]Shenzhen Institute of Artificial Intelligence and Robotics for Society, Shenzhen 518172, China.

[4]Department of Mechanical and Industrial Engineering, University of Toronto, Toronto ON M5S 3G8, Canada.

[5]School of Computer Engineering and Science, Shanghai University, Shanghai 200444, China.

[6]Department of Interventional Radiology, Chinese PLA General Hospital, Beijing 100853, China

[7]School of Medicine, The Chinese University of Hong Kong, Shenzhen 518172, China.

*Corresponding author: liuna_sia@shu.edu.cn, yujiangfan@cuhk.edu.cn

[+]These authors contributed equally to this work

The PDF file includes:

Supplementary Note 1 and Note 2
Supplementary Fig. 1 to Fig. 16
Supplementary Tables S1 and S2

Supplementary Methods

Supplementary Note 1 Analysis for translational locomotion of the robot

The magnetic multi-layer soft robot achieves translational motion on the gastric tissue by applying an external gradient magnetic field. The robot moves forward through translational motion actuated by magnetic force and the friction between the robot and the gastric tissue resists the robot's motion. The magnetic force is denoted as F_m and the friction force is denoted as F_f.

Calculation of magnetic field

We establish a cylindrical coordinate system with the center of the magnet as the origin O (Fig. 4a). In the translational motion of the robot, the magnetic field can be expressed as $\mathbf{B} = B_x\mathbf{e}_x + B_z\mathbf{e}_z$, where $\mathbf{e}_r$ and $\mathbf{e}_z$ are the positive unit vector of the x-axis and z-axis, respectively. B_x and B_z can be written as[1]:

$$B_x = -\frac{\mu_0}{4\pi}M\int_0^{2\pi}\int_0^{a}\left[-\frac{R(2r-2R\cos\phi)}{2(R^2+(L/2-z)^2+r^2-2Rr\cos\phi)^{3/2}}+\frac{R(2r-2R\cos\phi)}{2(R^2+(L/2+z)^2+r^2-2Rr\cos\phi)^{3/2}}\right]dRd\phi \quad (1)$$

$$B_z = -\frac{\mu_0}{4\pi}M\int_0^{2\pi}\int_0^{a}\left[\frac{R(L/2-z)}{(R^2+(L/2-z)^2+r^2-2Rr\cos\phi)^{3/2}}+\frac{R(L/2+z)}{(R^2+(L/2+z)^2+r^2-2Rr\cos\phi)^{3/2}}\right]dRd\phi \quad (2)$$

Here, (r,z) denotes the coordinates of the center point of the robot. μ_0 is the permeability of the vacuum. M is the scalar magnetization of the cylindrical permanent magnet. L and a are the thickness and radius of the magnet, respectively.

Calculation of the magnetic force

Additionally, we assume that the magnetization profile $\mathbf{m}$ perpendicular to its surface is uniformly distributed shown in Supplementary Fig. 1c. Therefore, the magnetic force exerted on the robot can be expressed as:

$$\mathbf{F}_m = V_m(\mathbf{m}\cdot\nabla)\mathbf{B} \quad (3)$$

where V_m represents the volume of the magnetic portion, and ∇ can be expressed as $\frac{\partial}{\partial x}\mathbf{e}_x + \frac{\partial}{\partial z}\mathbf{e}_z$. The decomposition of $\mathbf{F}_m$ into the x-axis and z-axis can be expressed as $\mathbf{F}_m = -F_m^x\mathbf{e}_x - F_m^z\mathbf{e}_z$, where F_m^x and F_m^z are both positive because the magnetic force is attractive between the magnet and the robot (Supplementary Fig. 3).

Calculation of the friction force

The friction force acting on the robot can be expressed as $F_f = \mu|F_N|$, given the normal force acting on it as F_N, where $\mu = \beta(p_z)^{\alpha-1}$ is the frictional coefficient with $\beta = 5.3$ and $\alpha = 0.54$. $p_z = |F_N|/s$ is the normal pressure[2].

Supplementary Note 2 Analysis of the tumbling motion of the robot

Analyzing the tumbling motion of the robot by setting the desired magnetic field $\mathbf{B}$. Firstly, the initial direction of the magnetic field is the positive y-axis direction (Fig. 4f and Supplementary Fig. 7). When $\mathbf{B}$ starts to rotate clockwise, the magnetic moment $\mathbf{m}$ tends to align with the direction of $\mathbf{B}$. The robot then achieves a tumbling motion actuated by the magnetic torque.

The magnetic field $\mathbf{B}$ can be expressed as:

$$\mathbf{B}(t) = B\begin{pmatrix} \sin(2\pi ft) \\ \cos(2\pi ft) \\ 0 \end{pmatrix} \tag{4}$$

where f represents the rotation frequency of the magnetic field.

The magnetic moment $\mathbf{m}$ can be expressed as:

$$\mathbf{m} = m\begin{pmatrix} 0 \\ 1 \\ 0 \end{pmatrix} \tag{5}$$

The magnetic torque τ_m can be expressed as:

$$\tau_m = -\begin{bmatrix} 0 & 0 & 1 \end{bmatrix}\{R\mathbf{m} \times \mathbf{B}\} \tag{6}$$

where R is the standard z-axis rotational matrix that accounts for the change in the direction of $\mathbf{m}$ due to the robot's flip:

$$R = \begin{bmatrix} \cos\varphi(t) & \sin\varphi(t) & 0 \\ -\sin\varphi(t) & \cos\varphi(t) & 0 \\ 0 & 0 & 1 \end{bmatrix} \tag{7}$$

where $\varphi(t)$ is the angular displacement of the robot.

Substituting Eq. (5), (6), and (7) into Eq. (4), τ_m can be written as:

$$\tau_m = mB\sin\left(2\pi ft - \varphi(t)\right) \tag{8}$$

As the robot tumbles, a supporting force F is provided at the contact surface (Supplementary Fig. 7). Assuming the centroid of the robot undergoes a parabolic motion, the direction of F is parallel to the motion direction of its centroid. Therefore, F can be expressed as:

$$F = M\dot{v}_f \tag{10}$$

where v_f represents the velocity of the centroid of the robot. Assuming that the robot does not slip as it rolls on the gastric tissue, the kinematic relationship between the v_f and $\varphi(t)$ can be expressed as:

$$v_f = \frac{l}{2}\dot{\varphi}(t) \tag{11}$$

Treating the tumbling motion as rotation around a fixed point, the conservation of momentum principle can be used to obtain:

$$\tau_m - C_f \dot{\varphi}(t) - F\frac{l}{2} = J\ddot{\varphi}(t) \tag{12}$$

The above equation can be rewritten as:

$$\dot{\varphi}(t) = \frac{mB\sin\left(2\pi ft - \varphi(t)\right) - \left(J + M\frac{l^2}{4}\right)\ddot{\varphi}(t)}{C_f} \tag{13}$$

where C_f, M, l, and J represent tumbling damping, the mass, length, and moment of inertia of the robot, respectively. Assuming that the robot does not slip during the tumbling process and can quickly reach a steady-state velocity under the influence of a magnetic field[3,4], Eq. (13) can be rewritten as:

$$\dot{\varphi}(t) = \frac{mB}{C_f}\sin\left(2\pi ft - \varphi(t)\right) \tag{14}$$

When the robot can achieve stable tumbling motion, the angle between the driving field and the angular displacement of the robot is constant, i.e., $\sin\left(2\pi ft - \varphi(t)\right)$ is a constant value. It can be seen that when the angle between the two is 90 degrees, i.e., $\sin\left(2\pi ft - \varphi(t)\right) = 1$, the robot can reach the maximum angular velocity. This maximum angular velocity is called the step-out frequency, which can be expressed as:

$$\dot{\varphi}_{step\text{-}out} = \frac{mB}{C_f}\sin\left(2\pi ft - \varphi\right) \tag{15}$$

It can be observed that once the rotation frequency of the magnetic field is higher than the step-out frequency of the robot, i.e., $2\pi f > \dot{\varphi}_{step\text{-}out}$, it becomes difficult to predict the angular velocity of the robot. At the same time, according to Eq. (15), it is found that in a stable tumbling motion, as the field strength increases, the step-out frequency of the robot increases.

Supplementary Figure

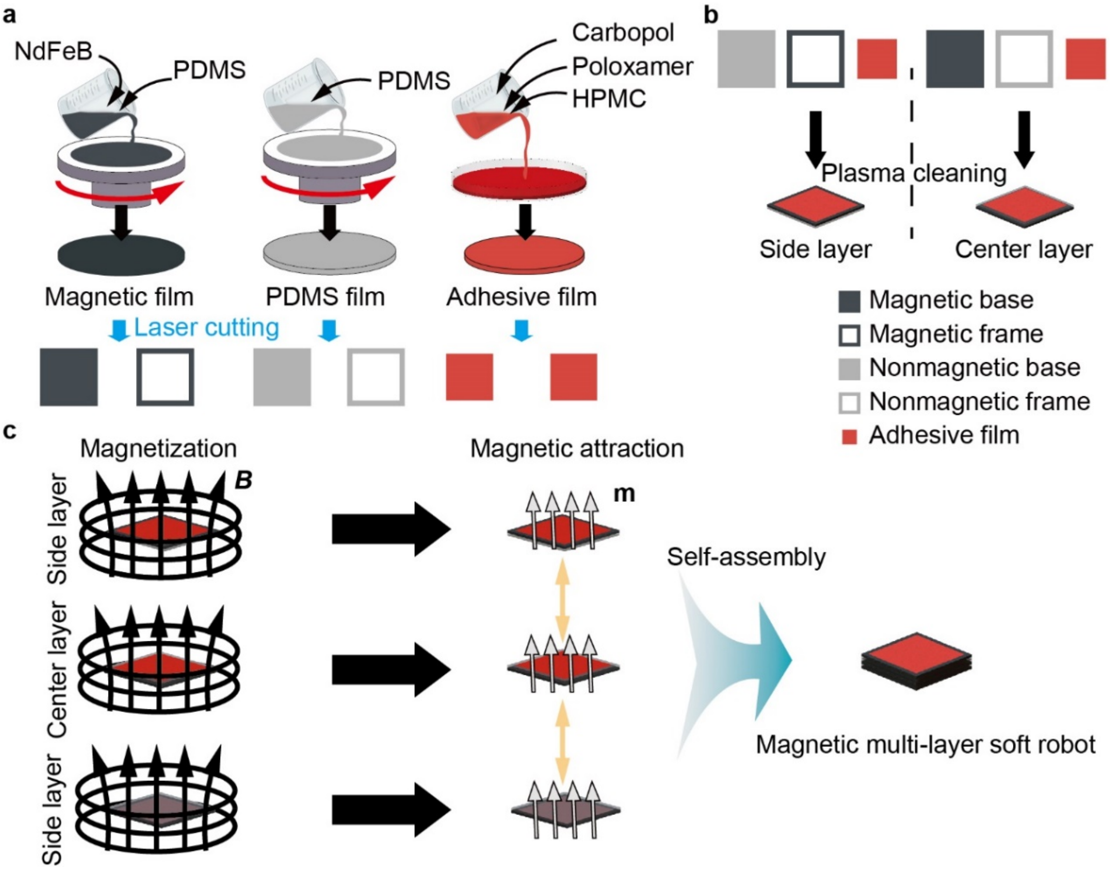

Supplementary Fig. 1 Fabrication of the magnetic multi-layer soft robot. **a**, Schematics of the fabrication steps of the components of the layers, including magnetic frame, magnetic base, nonmagnetic frame, nonmagnetic base, and adhesive film. **b**, Schematics of the fabrication of the layers. **c**, Schematics of fabrication of a magnetic multi-layer soft robot. Each layer is programmed same magnetization profile perpendicular to its surface by an electromagnet. The robot is then assembled through magnetic attraction.

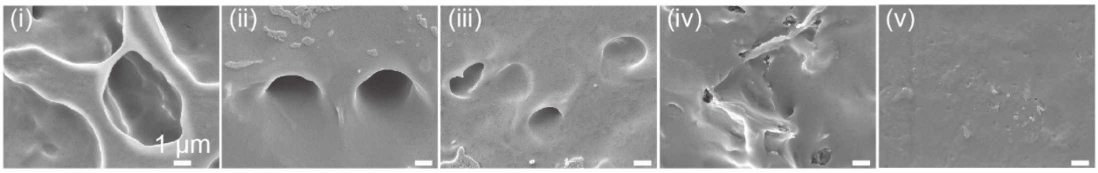

Supplementary Fig. 2 SEM of the adhesive films with different ratios of Carbopol : HPMC-Poloxamer mixture. (i) - (v) correspond to 1 : 6, 1 : 3, 1 : 2, 2 : 3, and 5 : 6 respectively.

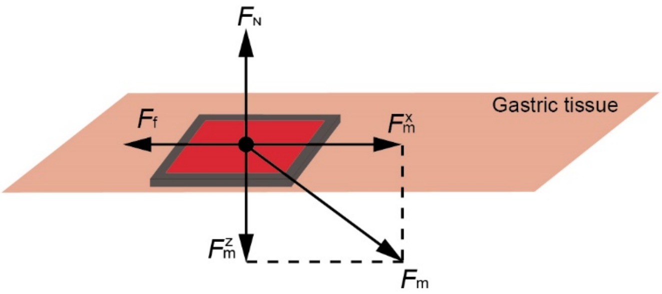

Supplementary Fig. 3 Schematics of the forces exerted on the robot when it performs the translational motion on the gastric tissue. The magnetic force is denoted as F_m and the friction force is denoted as F_f.

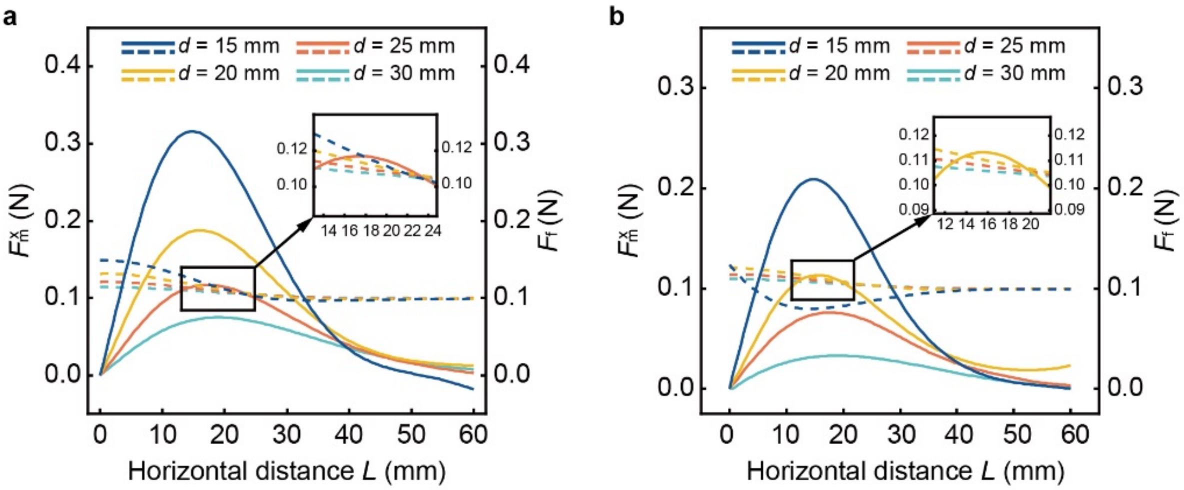

Supplementary Fig. 4 Simulation results and analysis of the forces exerted on the robot for its translational motion on gastric tissue. a-b, The relationship between the simulated superposed external force exerted on (**a**) a Type II robot and (**b**) a Type III robot performing translational motion and the horizontal distance L. Different vertical distances d are applied. The solid lines represent magnetic force F_m^r, and the dashed lines represent friction force F_f. Source data are provided as a Source Data file.

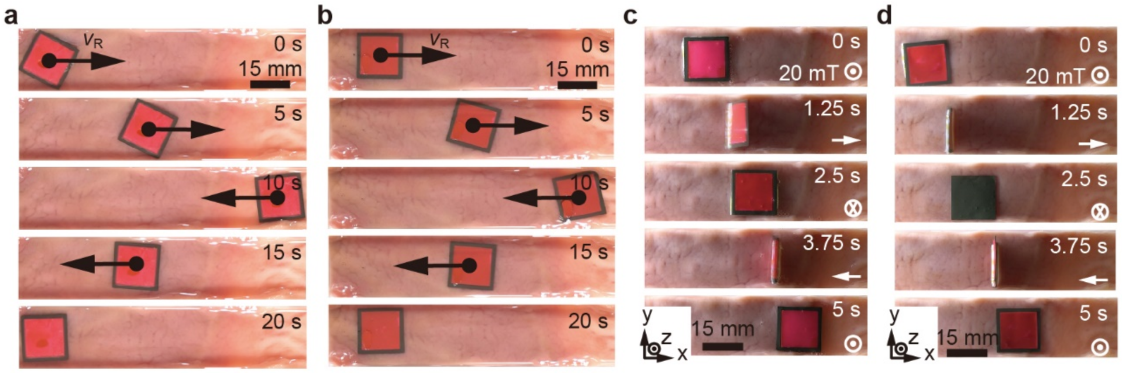

Supplementary Fig. 5 Locomotion of the robot on the gastric tissue. a-b, Translational motion of (**a**) a Type II robot and (**b**) a Type III robot on the gastric tissue. Tumbling motion of (**c**) a Type II robot and (**d**) a Type III robot on the gastric tissue.

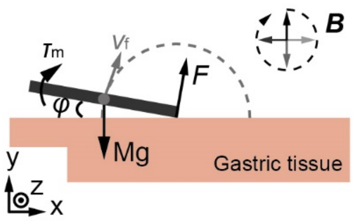

Supplementary Fig. 6 Schematics of the forces exerted on the robot when it performs the tumbling motion on the gastric tissue. The magnetic torque is denoted as τ_m, the gravity is denoted as Mg, and the supporting force is denoted as F.

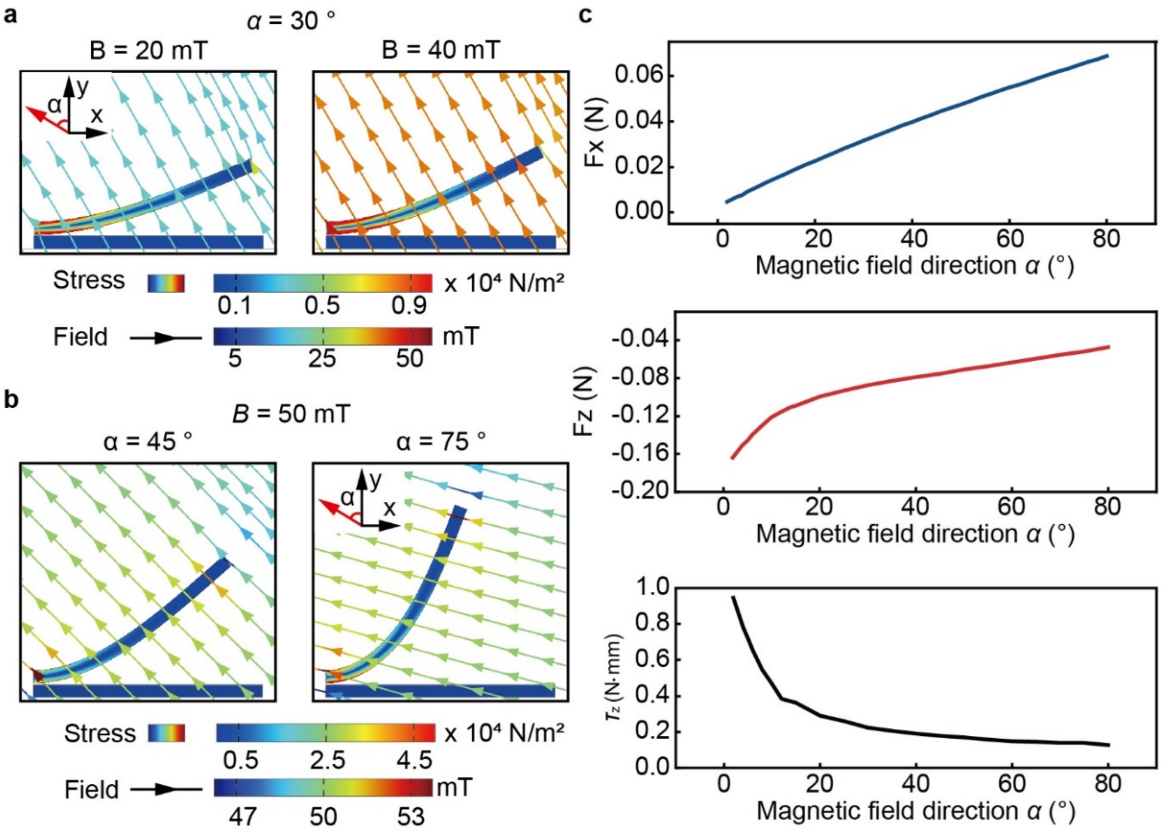

Supplementary Fig. 7 On-demand separation between the layers. a, Simulation of the separation process of a soft magnetic film actuated by a magnetic field with increased field strength (from 0 mT to 50 mT) and constant magnetic field direction. **b**, Simulation of the separation process of a soft magnetic film actuated by a magnetic field with changed field direction (from 0 ° to 90 °) and constant magnetic field strength. **c**, Simulation results of the layer-layer interaction forces and torques during separation process at B = 50 mT. Source data are provided as a Source Data file.

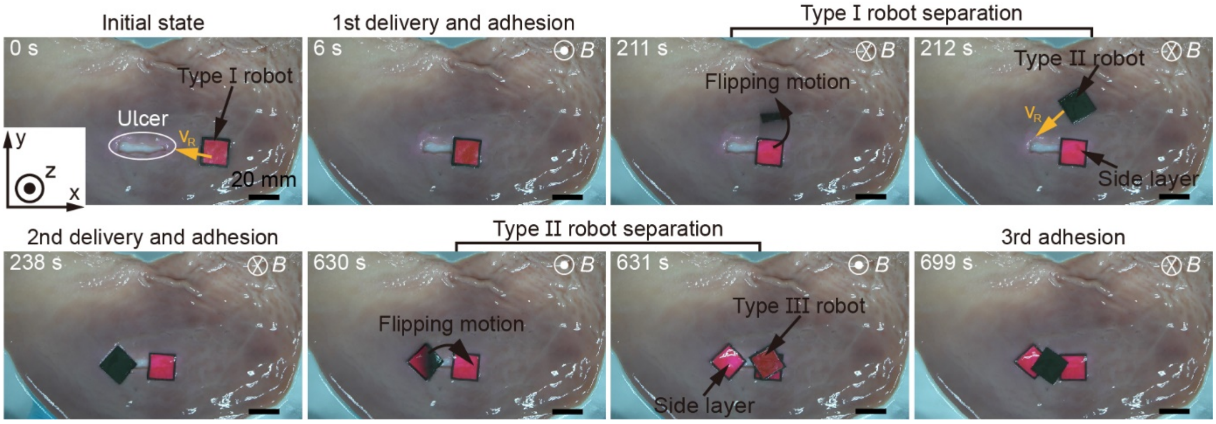

Supplementary Fig. 8 Experimental results of the multiple adhesion of the robot for larger ulcer on ex-vivo gastric tissue. The orange arrows represent the translational motion direction of the robots. The scale bar is 20 mm.

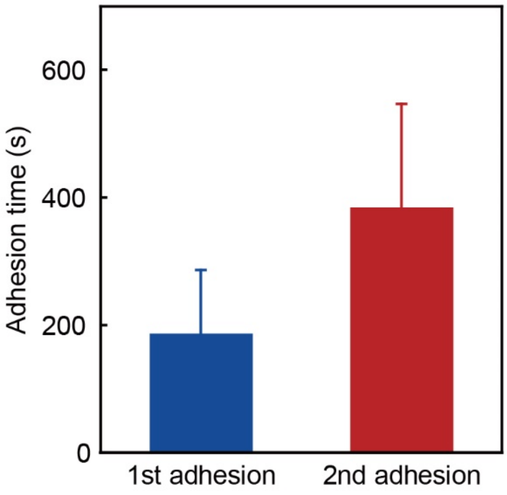

Supplementary Fig. 9 The indwelling time of adhesion formed between the side layer of the robot and the tissue. First adhesion formation is spent 184 ± 102 s, the second adhesion formation is spent 382 ± 164 s. Data are presented as mean values ± SEM. Source data are provided as a Source Data file.

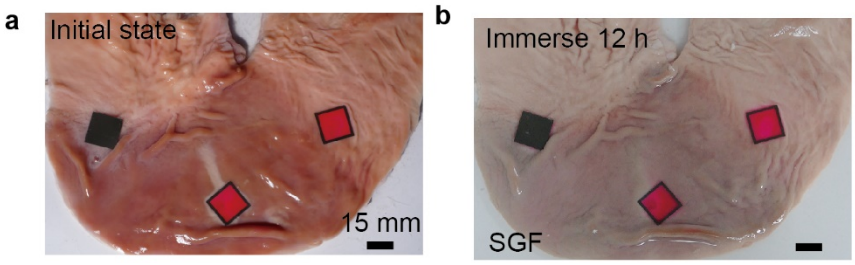

Supplementary Fig. 10 The retention performance of the side layer adhered to the ex-vivo tissue immersed in SGF at 12 h. a, Initial state of the gastric tissue with the layers of the robot. **b**, The layers of the robot remain adhered to the gastric tissue after immersing in SGF at 12 h.

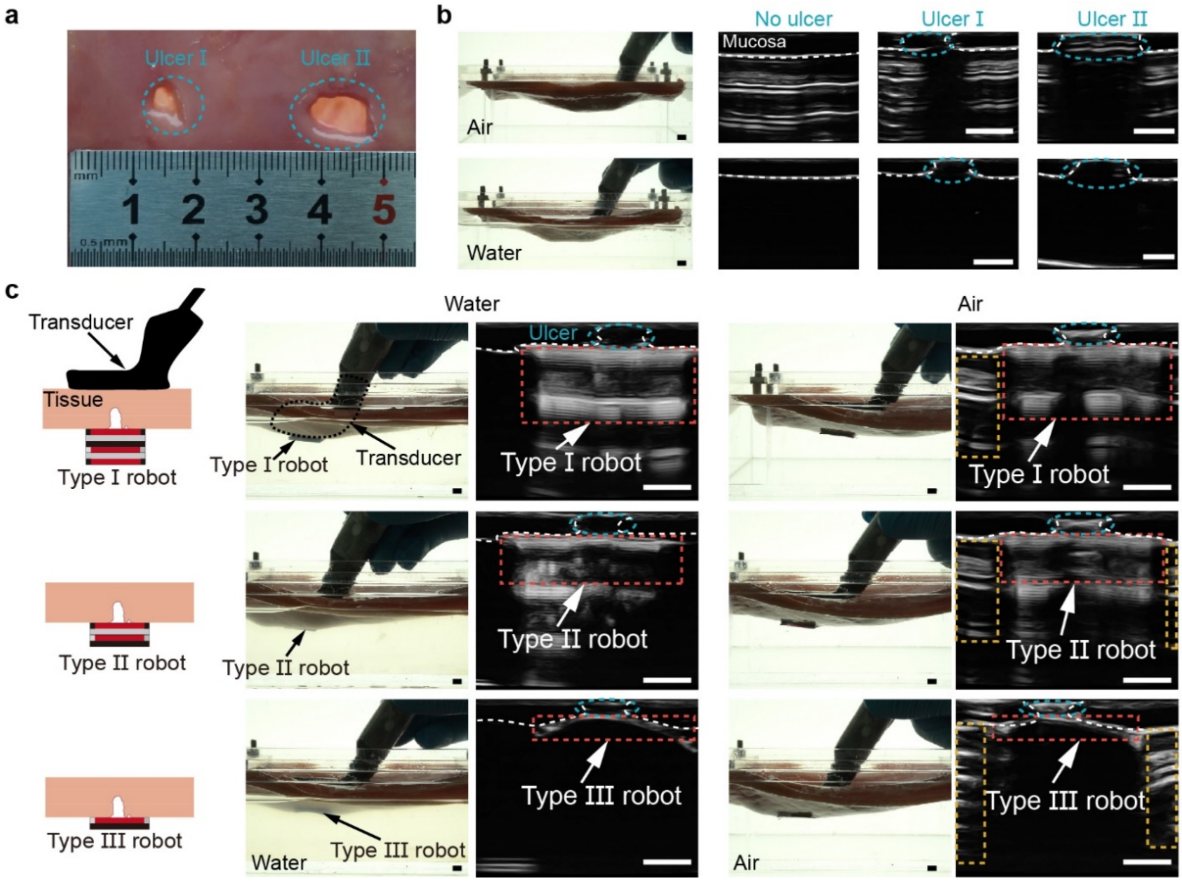

Supplementary Fig. 11 Characteristic ultrasound image of the robots and ulcers in different environments. a, Ulcers with different sizes on gastric tissue. **b**, Ultrasound images of the ulcers with different sizes in environments filled with water and air. The white dashed line and the blue dashed ellipses represent the gastric mucosa and the ulcer, respectively. The scale bar is 5 mm. **c**, Ultrasound images of the robot in environments filled with water and air. The blue dashed ellipses represent the gastric ulcer. The red dashed rectangles represent the robot. The yellow dashed rectangles represent white signal. The scale bar is 5 mm.

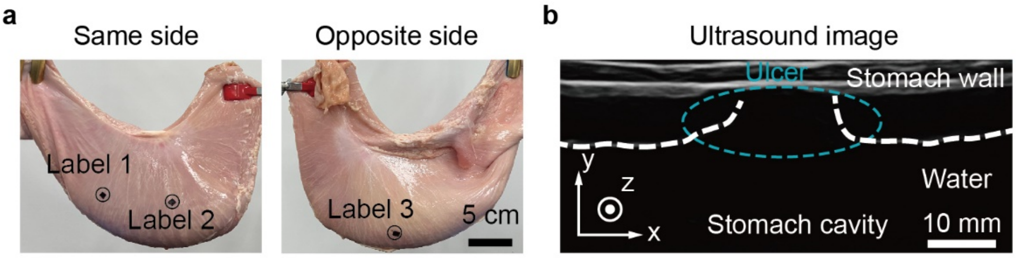

Supplementary Fig. 12 Experimental setup of the ex vivo porcine stomach. a, Three ulcers are prepared in the porcine stomach, distributed on both sides of the stomach. **b**, Ultrasound image of an ulcer inside of the ex-vivo stomach. The white dashed line represents the mucosa.

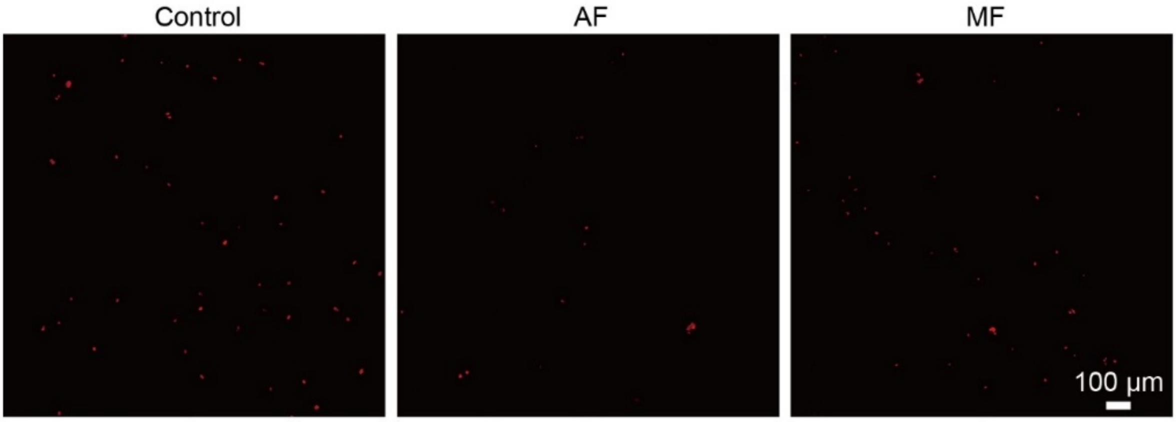

Supplementary Fig. 13 Live/dead staining image of GES-1 cells in three treatment groups after 24-hour co-culture.

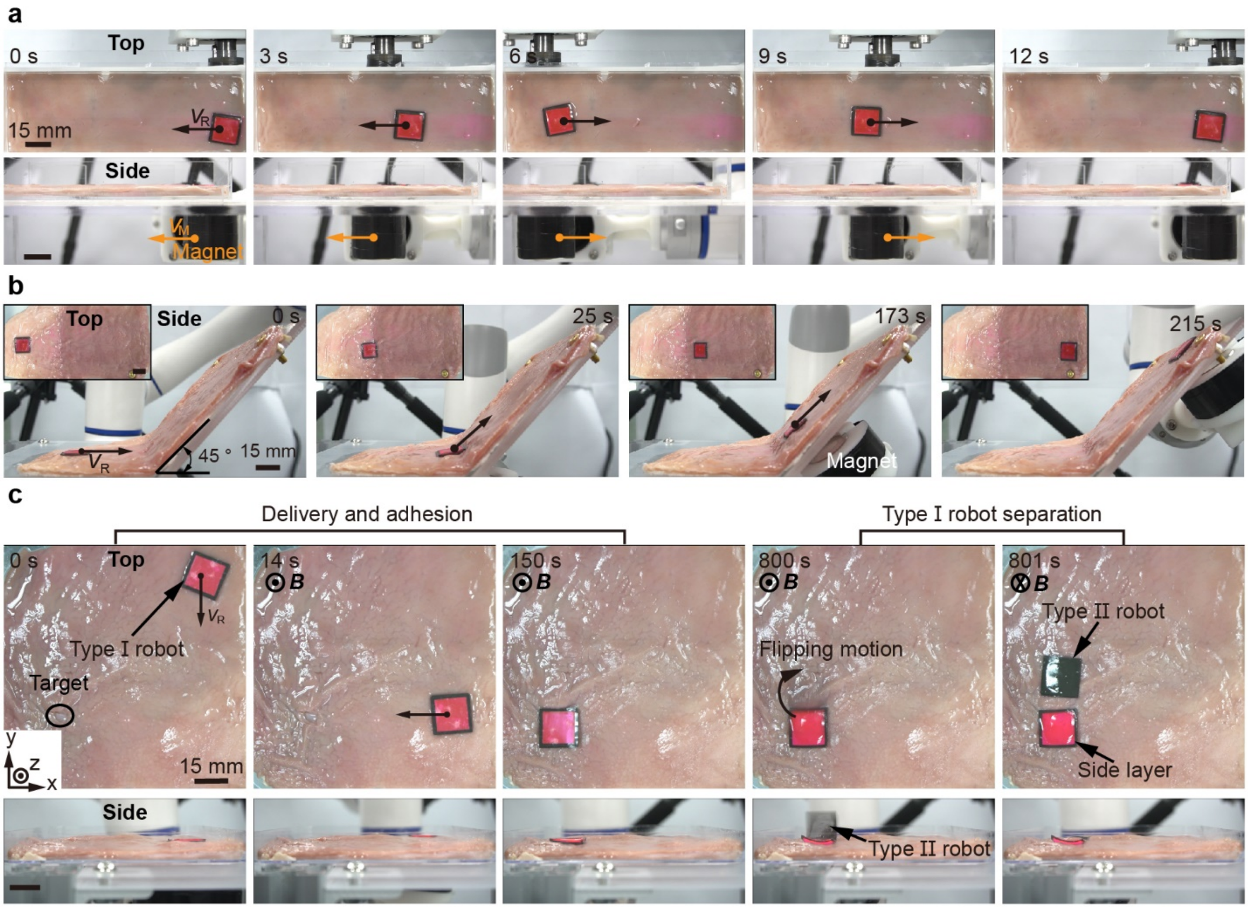

Supplementary Fig. 14 Locomotion of the robot operated by a robot arm with a magnet. **a**, Translational motion of the robot. The orange arrows represent the translational motion direction of the magnet. **b**, Climbing motion of the robot on a 45° slope. **c**, Targeted navigation and separation of the robot from the adhered side layer.

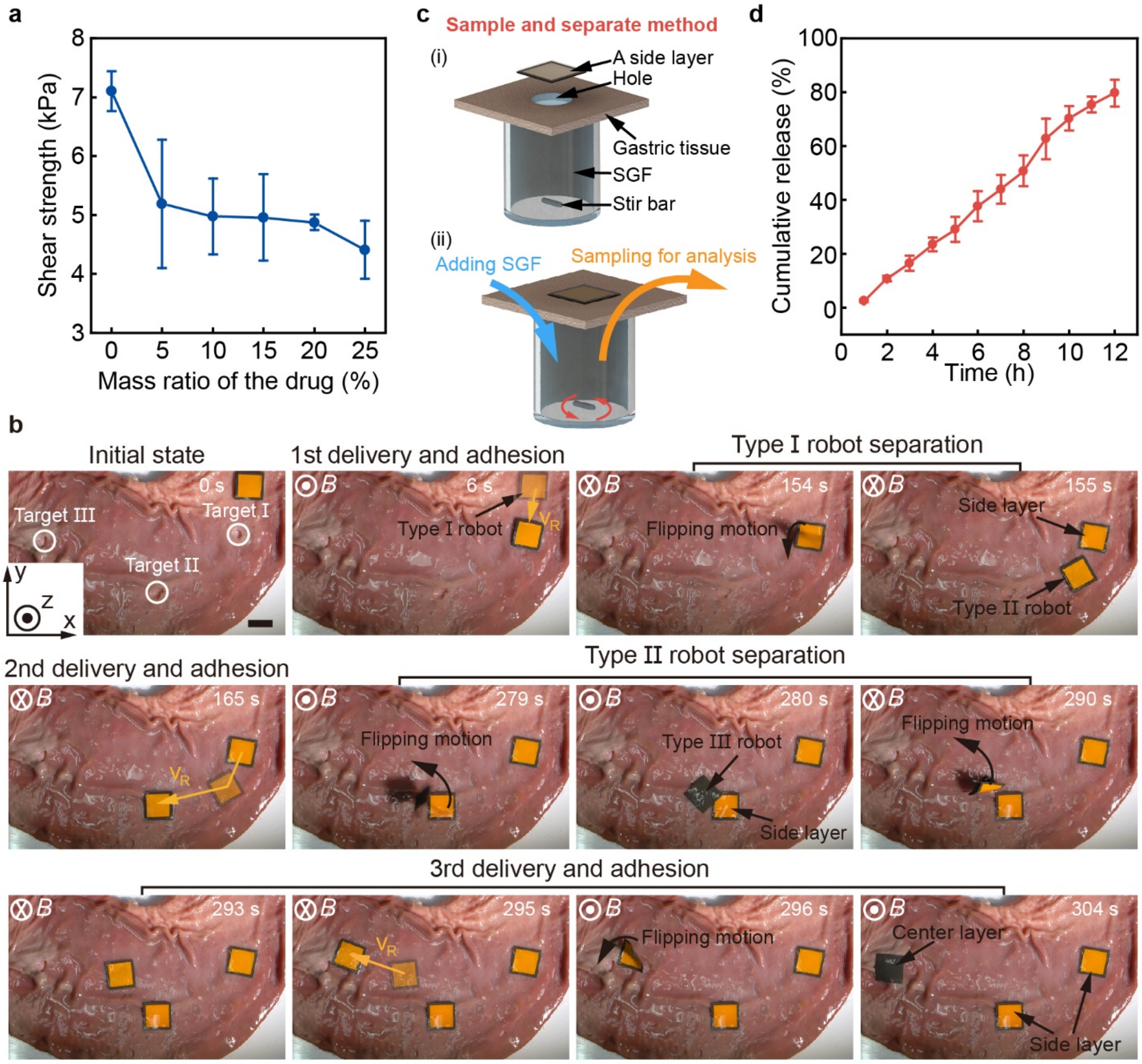

Supplementary Fig. 15 Characterization of adhesion performance and drug release of the drug-loaded adhesive films. **a**, Relationship between shear strength of the adhesive films and the drug-loaded ratio. The error bars are obtained from 3 trials in each condition. **b**, Experimental results of the multi-target adhesion using the robot loaded 25 % curcumin on ex-vivo gastric tissue. The scale bar is 15 mm. **c**, Schematics of sample and separate method. **d**, Cumulative release of curcumin loaded by the adhesive film. The error bars are obtained from 3 trials in each condition. Data are presented as mean values ± SEM. Source data are provided as a Source Data file.

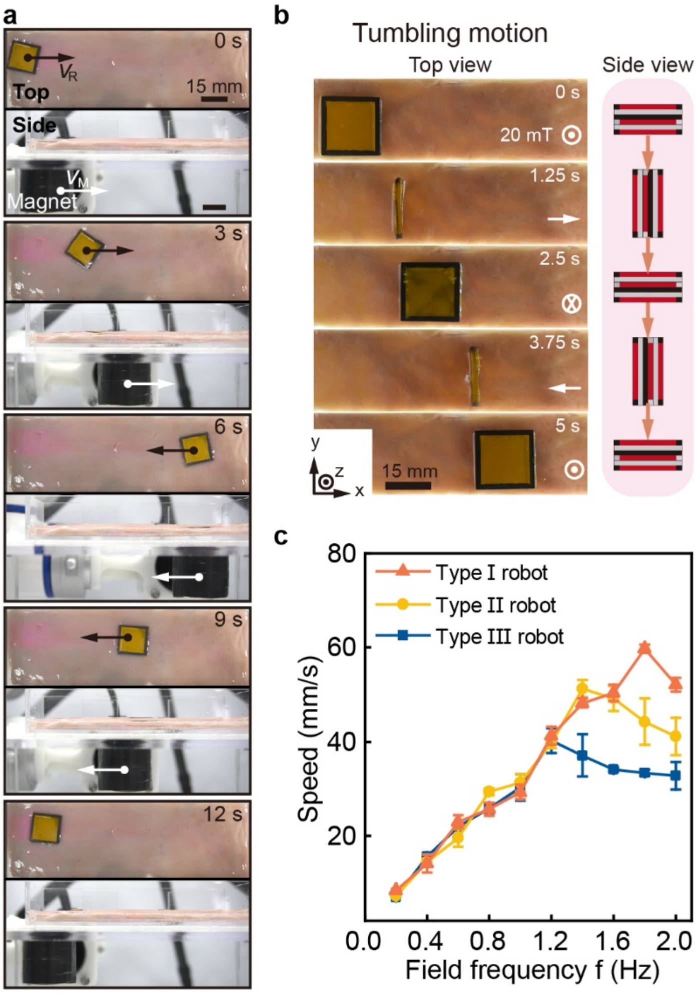

Supplementary Fig. 16 Locomotion of the drug-loaded robots. a, Translational motion of the robot on the gastric tissue at a vertical distance of 20 mm. The white arrows represent the translational motion direction of the magnet. **b**, Tumbling motion of the robot on gastric tissue. **c**, Relationship between the speed of the robot performing tumbling motion and the frequency of the magnetic field. The error bars are obtained from 3 trials in each condition. Data are presented as mean values ± SEM. Source data are provided as a Source Data file.

Supplementary Table S1

The effective range of the horizontal distance L, at which the magnetic multi-layer soft robot can be actuated on the gastric tissue. Different vertical distances d are applied.

	Horizontal distance L /mm			
	d = 15 mm	d = 20 mm	d = 25 mm	d = 30 mm
Type I Robot	$3.5 < L < 36$	$5.5 < L < 33.5$	$10 < L < 29$	/
Type II Robot	$4.5 < L < 33$	$7.5 < L < 30.5$	$14.5 < L < 23$	/
Type III Robot	$5 < L < 29$	$14 < L < 19.5$	/	/

Supplementary Table S2

Detailed information of the proposed robot.

	Type I robot	Type II robot	Type III robot
Weight (g)	0.612 ± 0.054	0.412 ± 0.025	0.221 ± 0.007
Length × Width × Thickness (mm^3) of the robot	18 × 18 × 1.5	18 × 18 × 1.1	18 × 18 × 0.7
Length × Width × Thickness (mm^3) of adhesive film	15 × 15 × 0.08	15 × 15 × 0.08	15 × 15 × 0.08

Supplementary Reference

1. S.M., B. Magnetic Field of a Cylindrical Bar Magnet. http//demonstrations.wolfram.com/Magnetic Field Of A Cylindrical Bar Magnet/.
2. Liu, X. *et al.* Magnetic Living Hydrogels for Intestinal Localization, Retention, and Diagnosis. *Adv. Funct. Mater.* **31**, 2010918 (2021).
3. Diller, E., Giltinan, J. & Sitti, M. Independent control of multiple magnetic microrobots in three dimensions. *Int. J. Robot. Res.* **32**, 614–631 (2013).
4. Mahoney, A. W., Nelson, N. D., Peyer, K. E., Nelson, B. J. & Abbott, J. J. Behavior of rotating magnetic microrobots above the step-out frequency with application to control of multi-microrobot systems. *Appl. Phys. Lett.* **104**, 144101 (2014).
