# Supplementary material for: A magnetic multi-layer soft robot for on-demand targeted adhesion
Source: Nat Commun. 2024 Jan 20;15:644. doi: 10.1038/s41467-024-44995-9 (PMC10799857; doi:10.1038/s41467-024-44995-9)
Supplement: Supplementary file 3 — Description of Additional Supplementary Files [file 41467_2024_44995_MOESM3_ESM.pdf]

Captions for Supplementary Movies

Supplementary Movie 1: On-demand separation of the robot

This video sequentially shows the on-demand separation of the robot (Corresponding to Fig. 5d).

Supplementary Movie 2: Multi-target adhesion on porcine gastric tissue

This video sequentially shows the multi-target adhesion using the robot on ex-vivo gastric tissue (Corresponding to Fig. 6b).

Supplementary Movie 3: Multi-target adhesion in an ex-vivo porcine stomach

This video sequentially shows the multi-target adhesion using the robot in an ex-vivo porcine stomach under real-time tracking of ultrasound imaging (Corresponding to Fig. 7a).

Supplementary Movie 4: Multi-target adhesion in an in-vivo porcine stomach

This video sequentially shows the real-time navigation of a robot in an in-vivo porcine stomach for multi-target adhesion (Corresponding to Fig. 8b).
